# Upjohn Pill and Granule Co.

**Published:** 1889-07

**Authors:** 


THE UPJOHN PILL AND GRANULE Co, have capped the climax in producing a coating for pills, or rather powders, for on rupturing the thin pelicle or coating to their pills the drug will be found pure, dry and finely pulverized. See their interesting advertisement in this Journal.

